# Anatomical variations of the sternum: sternal foramen and variant xiphoid morphology in dried adult human sternum in Ethiopia

**DOI:** 10.12688/f1000research.133151.1

**Published:** 2023-08-07

**Authors:** Alemayehu Shiferaw Lema

**Affiliations:** 1 Department of Forensic Medicine and Toxicology, St. Paul's Hospital Millennium Medical College, Addis Ababa, Ethiopia

**Keywords:** Sternum, Sternal foramen, Xiphoid morphology, Variations, Sternal puncture, Acupuncture, Forensic, Radiology, Ethiopia

## Abstract

**Background:** The sternum exhibits unique anatomical variations with major clinical and forensic implications. This study is devoted to providing baseline epidemiological information about the sternal foramen and variant xiphoid morphology in Ethiopia. Two extremely interesting and unusual variations of the sternal foramen are also discussed.

**Methods:** This observational study was carried out using dried adult human sternum obtained from skeletal remains samples brought for medicolegal examination over a period of 4 years. A total of 94 dried adult human sternums (66 males (70.2%) and 28 females (29.8%)) were obtained with an age range of 21 to 57 years and a mean age at death of 38.383 ± 11.3480 years. Dried human sternum specimens were morphologically examined, and morphometric parameters were recorded and photographed.

**Results:** A sternal foramen was found in 18 specimens (19.1%); 17 were male and one was female. A single sternal foramen was observed in 83.3% (n=15/18) of the sternal bodies and 11.1% (n=2/18) of the xiphoid processes (both males). In addition, a double sternal foramen was observed in a single male specimen on the mesosternum and xiphoid process. The most common sternal foramen site was at the fifth costochondral junction level. The xiphoid process was present in 77 samples and ended as a single process in 83.1% (n=64/77) of samples. In 15.6% (n=12/77) of the samples, the xiphoid process was bifurcated and trifurcated in a single male (1.3%) specimen.

**Conclusions:** The sternal foramen and variation in xiphoid morphology are common anatomical variations in Ethiopia. The findings of the current study highlight the necessity of strict precautionary measures during sternal procedures in this study population. In addition, such incidental findings during radiologic and autopsy procedures should be properly evaluated to avoid misdiagnosis and misinterpretation of such findings as traumatic or pathologic conditions.

## Introduction

The human sternum forms the anterior midline border of the thorax and is located adjacent to the pleura cavity, pericardial cavity and great vessels of the thoracic cavity. ^1^ The human sternum comprises three components: the manubrium, the mesosternum (body), and the xiphoid process. ^1^^,^^2^ Human skeletons have several variations that may require a distinction from pathologic alterations. The sternum is one of the parts of the human skeleton with the highest frequency of anatomical variation. ^2^

Sternal foramen (SF) is a round or oval or even irregular bony defect in any part of the sternum that results from the incomplete union of the mesenchymal sternal bars during embryogenesis. ^2^^–^^4^ SFs are usually asymptomatic and are frequently detected incidentally in radiological images, postmortem examinations and anatomy teaching specimens. ^2^^–^^6^ Knowledge of congenital SF has various implications in clinical, acupuncture, and forensic practices. ^2^^–^^6^

Although SF is asymptomatic, its proximity to vital thoracic structures may pose a serious hazard during sternal procedures. ^2^^,^^3^^,^^5^ In addition, SF can be misdiagnosed as lytic lesions or trauma in the interpretation of radiologic images and postmortem findings of skeletonized human remains. ^2^^,^^4^^–^^6^ Therefore, awareness concerning sternal bone variations is important for radiologists and forensic experts to avoid misinterpretation.

Moreover, sternal bone variation analysis is a simple and important potential source of data in the human skeleton identification process whenever there is recorded variation in the antemortem data of the victim. ^2^^–^^4^ SFs have been detected in the manubrium, mesosternum and xiphoid process; however, they are more frequently observed in the lower portion of the sternum. The incidence of sternal foramina varies between different populations, ranging from 3.1 to 18.3%. ^1^^,^^2^^,^^4^^,^^5^^,^^7^^–^^18^

On the other hand, the xiphoid process is the most variable portion of the sternal bone. ^5^ Although educational textbooks describe the xiphoid process as pointed and elongated, it can be absent, broad, bifid, trifurcated, duplicated, or deflected and may contain a foramen. Such variations could be misinterpreted as an epigastric mass, fracture or traumatic fissure. ^1^^,^^5^

Although the distribution of these sternal variations shows discrepancy among different populations, data from Africans are scarcely reported. To my knowledge, no study has described sternal variations in the Ethiopian population. This study is devoted to providing baseline epidemiological information about the sternal foramen and variant xiphoid morphology in Ethiopia, comparing results with other populations and discussing the clinical and forensic significance.

## Methods

This observational study was carried out using dried adult human sternum obtained from skeletal remains samples brought for medicolegal examination from January 1, 2019, to December 31, 2022, in the Department of Forensic Medicine and Toxicology, St. Paul’s Hospital and Millennium Medical College in Addis Ababa, Ethiopia. All skeletal remains samples obtained during the study period of both sex and adult age groups that had human sternal bone were included in this study. Brocken sternal bone specimens and those without sex or age records were excluded from the study. Dried human sternum specimens were morphologically examined for the presence of SF, and their topology and morphometric parameters were recorded and photographed. The xiphoid process was classified as absent or present, and its terminal end was classified as single, bifurcated, or trifurcated. Samples were photographed, and SF measurements were obtained using a Vernier caliper with an accuracy of 0.02 mm. Data were analyzed using SPSS version 26.0. Continuous data were described in terms of the mean and compared using the Mann–Whitney U test. Categorical variables were described using frequencies and percentages and compared using the chi-square test. The level of significance was set at a p-value <0.05. The study was conducted as per the Declaration of Helsinki. Ethics approval was obtained from the St. Paul’s Hospital and Millennium Medical College institutional review board (ethical clearance reference no: PM 23/392). All information was treated anonymously and confidentially using unique identification codes rather than individual names and identifications. Waiver of informed consent was granted by the institutional review board, because of the anonymization of the data, the difficulty of the reaching next-of-kin during the examination, the potential value that the findings may add to clinical and forensic practices and the low risk involved in the use of the data.

## Results

A total of 94 dried adult sternum specimens were available for the study. The 94 dried adult sterna samples examined constituted 66 males (70.2%) and 28 females (29.8%). SF was observed in 18 specimens, resulting in an incidence of 19.1% of the specimen. Among the 18 specimens with SF, 17 specimens (94.4%) were male, and a single specimen (5.6%) was female. There was a statistically significant difference between the presence of SF and sex (p=0.012). Ages at death ranged from 21 to 57 years, and the mean age at death was 38.383±11.3480 years. The sternal foramen was more frequent (61.1%) in individuals over 35 years of age, and there was a statistically significant difference between the presence and absence of the sternal foramen between younger individuals (<35 years) and older (≥35 years) individuals (p<0.001). However, its presence was not different in the younger (<35 years) and older (≥35 years) age groups at death in the pooled sex sample (p=0.250).

**Figure 1.**
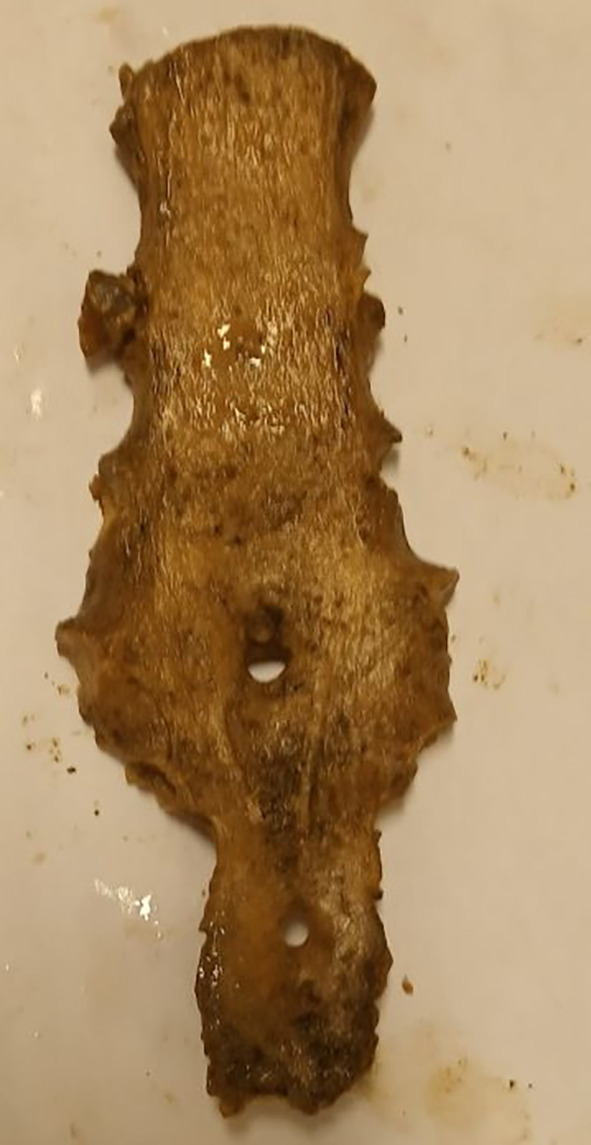
Dried sternal specimen with double foramen at the body and xiphoid process.

**Figure 2.**
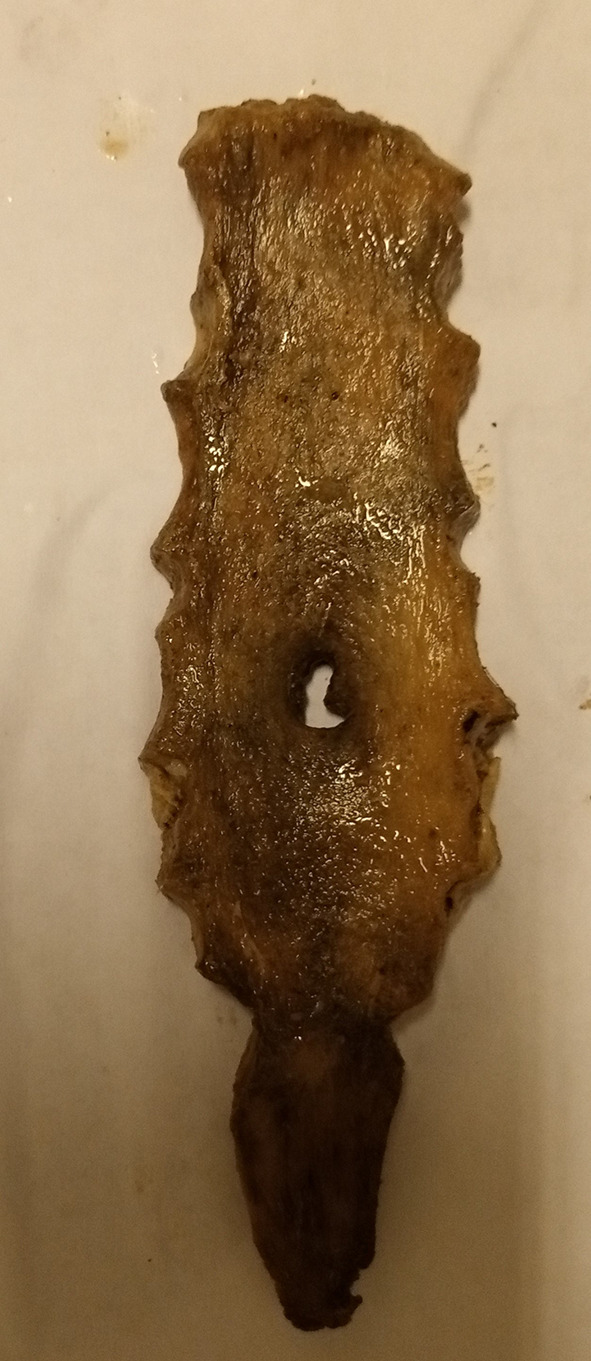
Dried sternal specimen with irregular margin and shape.

**Figure 3.**
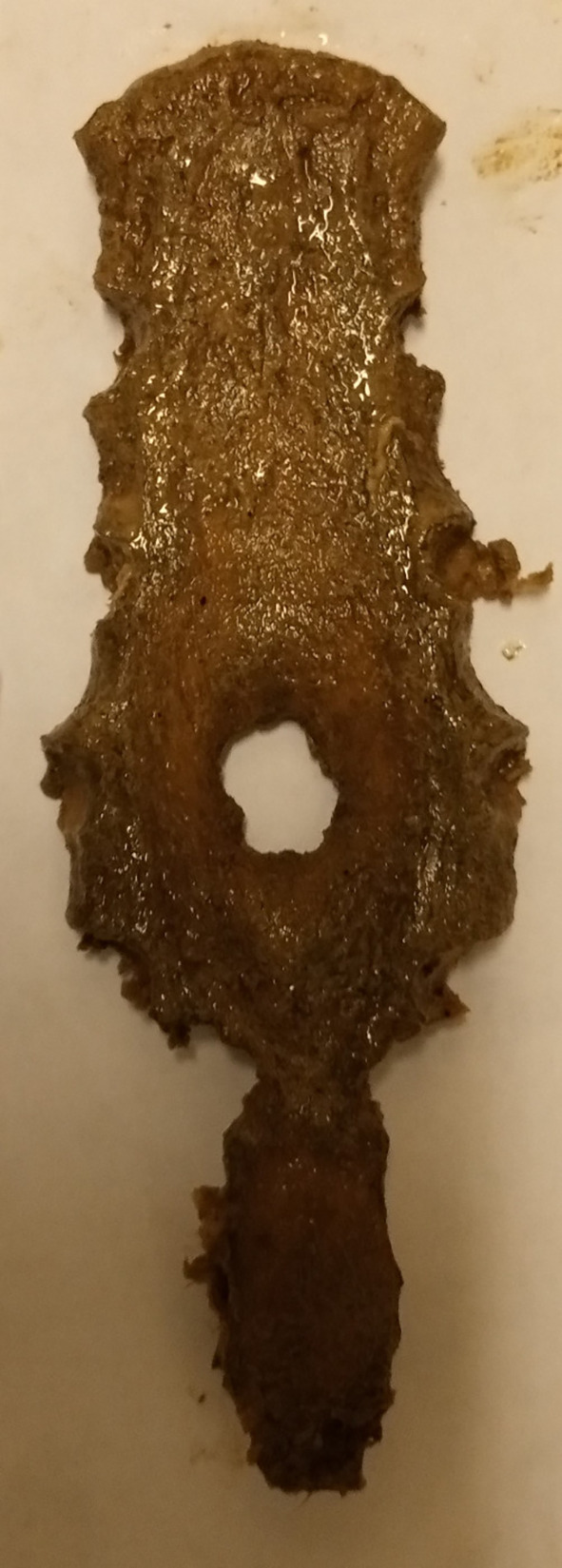
Dried sternal specimen with irregular margin and abnormal shape.

**Figure 4.**
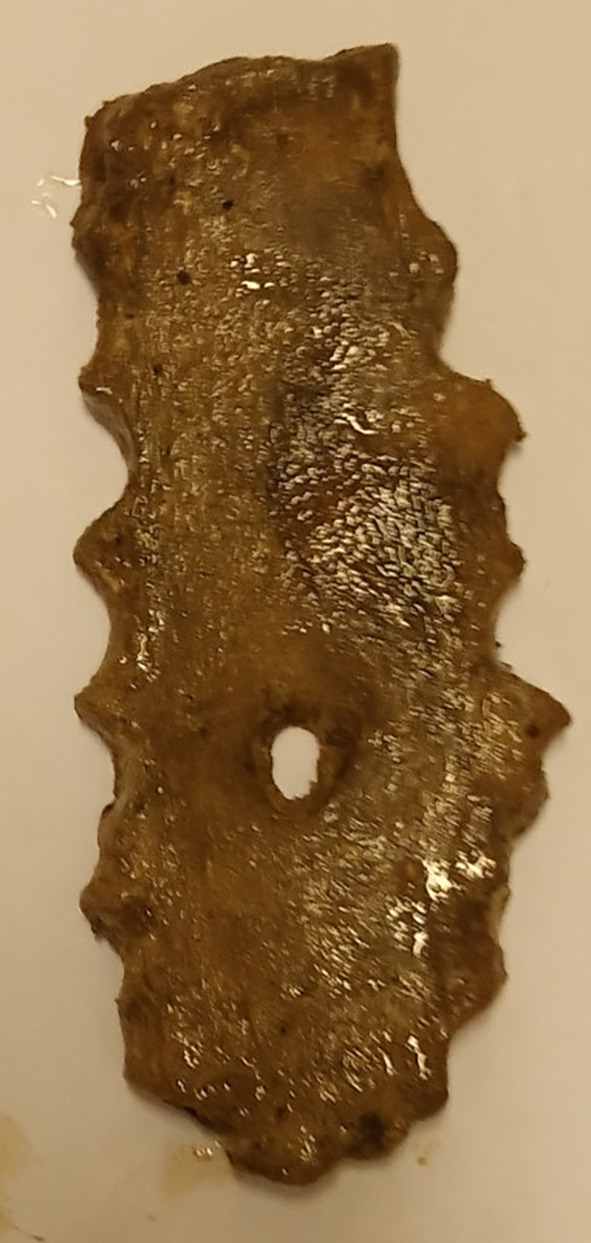
Dried sternal specimen with irregular and beveled margin.

A single SF was observed in the sternal body in 83.3% (n=15/18), while two (11.1%) single SF were detected in the xiphoid process (both males). In addition, a double sternal foramen was observed in a single male specimen on the sternal body and xiphoid process ( Figure 1). However, there was no statistically significant difference between the topology of the SF (sternal body and xiphoid process) and sex (p=0.813). No foramen was observed on the manubrium or upper mesosternal segments proximal to the level of the fourth costochondral junction (CCJ). The most common site of SF was at the level of the fifth costochondral junction (CCJ) in 55.6% (n=10/18). This is followed by the fourth CCJ, sixth CCJ and xiphoid process, where SF is observed in 16.7% (n=3/18) of specimens at each level. However, there was no statistically significant difference between the topology of SF and both the vertical length (P=0.159) and transverse width (P=0.054) of SF. Four SFs (22.2% (n=4/18) with irregular bony margins and abnormal shapes were observed on the body of the sternum ( Figures 2, 3 and 4). A single case of SF with a beveled margin was also documented ( Figure 4). The mean transverse width of the SF was 7.08±3.325 mm (3-16 mm), and the vertical length was 7.88±4.285 mm (3-18 mm).

**Figure 5.**
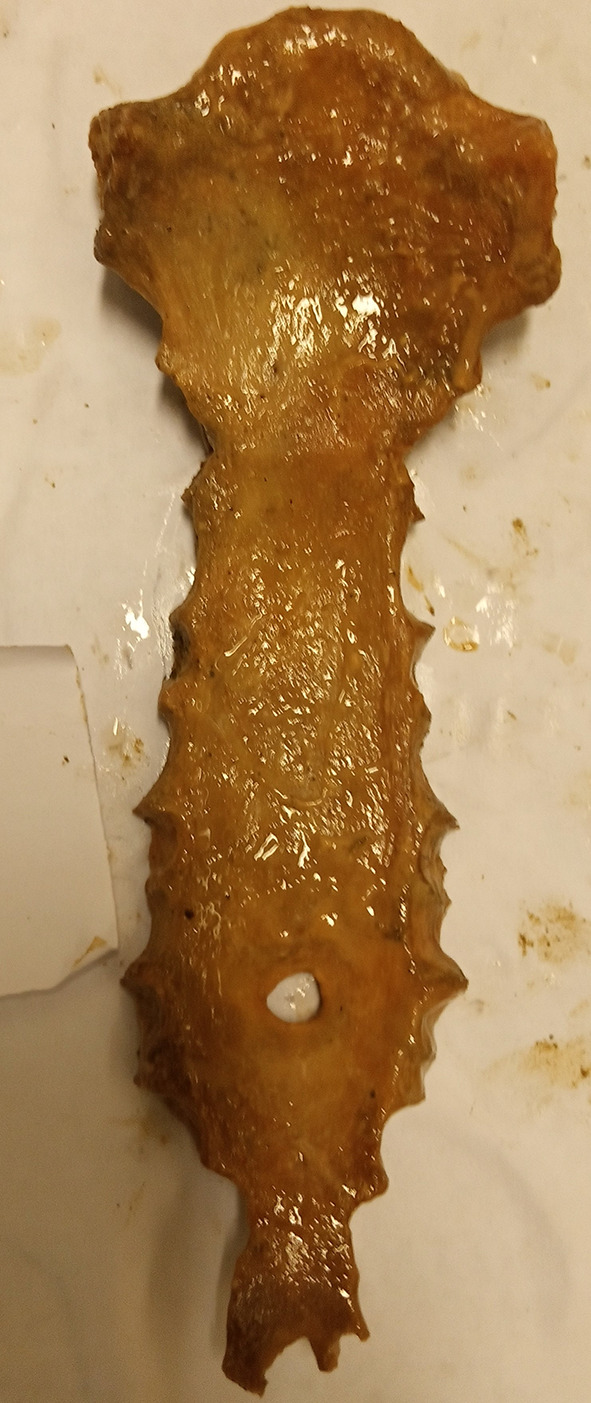
Dried sternal specimen with the trifurcated xiphoid process end.

The xiphoid process was absent in 18.1% of specimens, including 13.8% males (n=13) and 4.3% females (n=4), and the majority (84%) of these individuals were below 30 years of age. There was no statistically significant difference between the presence and absence of the xiphoid process between younger individuals (<50 years) and older (>50 years) individuals (p=0.178) and sex (p=0.533). The xiphoid process ended as a single in 83.1% (n=64/77) of specimens, including 58.4% (n= 45) males and 24.7% (n=19) females. In 15.6% (n=12/77) of the samples, the xiphoid process was bifurcated and trifurcated in a single male (1.3%) specimen ( Figure 5). However, no differences were observed between variations in xiphoid morphology with sex (p=0.670) or the presence of the sternal foramen (p=0.157).

## Discussion

### Embryology

The sternum develops from mesenchymal condensation that forms paired sternal bars that were originally located on each side of the midline. Immediately after the attachment of the cranial ribs, these paired sternal bars begin to chondrify, migrate to the midline and fuse in a craniocaudal fashion. After chondrification, ossification centers begin to appear from the fifth month to early childhood in craniocaudal succession. These ossification centers in the body of the sternum develop into four transverse segments known as the mesosternum. The ossification centers for the mesosternal segments vary considerably. In general, the two proximal mesosternal segments have single centers, whereas the third and fourth segments have paired centers. Beginning in the third to the fourth year of life, the mesosternal segments fuse caudocranially and are typically completed in early adulthood. ^19^^,^^20^ During childhood, single or paired ossification centers appear in the xiphoid process. ^4^ Because of the presence of paired ossification centers in the lower portion of the sternal bone (mainly the lower mesosternal segments and the xiphoid process), the lower parts are the most variable portion of the sternal bone. Occasionally, the fusion of the paired ossification centers fails across their midline, resulting in a sternal foramen. ^19^ There may be an incomplete fusion of the lower end of the sternum, resulting in a trifurcated or bifurcated xiphoid process. ^5^

### Epidemiology and topology

The incidence of SF varies in different studies from different populations and reported incidence data on SF from different populations ranging from 3.1 to 18.3%. ^1^^,^^2^^,^^4^^,^^5^^,^^7^^–^^18^ Data from the literature on the incidence of SF are summarized in Table 1. The findings of the current study reveal that the incidence of SF in the Ethiopian population is 19.1%. This is inconsistent with many reported studies from American and European populations. ^8^^–^^10^^,^^14^^,^^15^ Previous studies in Brazil, Greece, and East Africa documented a higher incidence. which is in agreement, although slightly lower than our finding. ^1^^,^^4^^,^^5^^,^^7^^,^^11^ The variation in the reported incidence of SF could be due to geographic ethnic, racial, and genetic differences. This finding indicates that SF is a common congenital variation in Ethiopia and highlights the need for strict precautionary measures during sternal procedures in this study population. In addition, such incidental findings during radiologic and autopsy procedures should be properly evaluated to avoid misdiagnosis and misinterpretation of such findings as traumatic or pathological conditions. ^1^^,^^2^^,^^4^^,^^5^

**Table 1.** Comparison of the incidence of sternal foramina found in the cited literature.

Authors (Year)	Population	Type of sample (Specimen)	Incidence of SF (%)
Ashley (1956) ^7^	East Africans	Anatomic (Dry Specimens)	13.3
	Europeans	Anatomic (Dry Specimens)	4
Cooper (1988) ^8^	USA	Cadaveric (X-ray)	6.7
Schratter (1997) ^9^	German	Radiologic (CT)	6
Yekeler (2006) ^10^	Turkish	Radiologic (MDCT)	4.5
Babinski (2012) ^11^	Brazilian	Cadaveric (Dry Specimens)	16.6
El- Bushaid (2012) ^5^	Kenyans	Anatomic (Dry Specimens)	13.8
Ishii (2012) ^12^	Japanese	Radiologic (MDCT)	3.1
Singh (2013) ^13^	India	Cadaveric (Dry Specimens)	11.9
Macaluso (2014) ^14^	Spanish	Radiologic (X-ray)	3.3
Bayarogullari (2014) ^15^	Turkish	Radiologic (MDCT)	6
Paraskevas (2015) ^4^	Greek	Anatomic (Dry Specimens)	18.3
Babinski (2015) ^2^	Brazilian	Radiologic (MDCT)	10.5
Boruah (2016) ^16^	India	Radiologic (MDCT)	11.6
Kirum(2017) ^1^	Uganda	Anatomic (Dry Specimens)	12.9
Kuzucuoglu (2020) ^17^	Turkish	Radiologic (CT)	8.44
Bolatli (2020) ^18^	Australia	Radiologic (MDCT)	16.8
Present Study (2023)	Ethiopian	Anatomic (Dry Specimens)	19.1

Consistent with previous studies, significant differences were observed in which sternal foramina were more common in men than in women. ^3^^,^^6^ In the current study, no foramen was observed in the manubrium or upper mesosternal segments proximal to the level of the fourth CCJ. The most common site of SF was at the level of the fifth CCJ, accounting for more than half of the specimens with SF. This is followed by the fourth CCJ, sixth CCJ and xiphoid process, where SF is observed in 3 specimens at each site. Double sternal foramen was observed in a single male specimen on the sternal body and xiphoid process. This is consistent with a study from Kenya in which SFs were predominantly observed in the lower sternal segments, but differed from studies from Turkey and Greece that revealed more SFs in the xiphoid process than in the lower mesosternal segments. ^4^^,^^5^^,^^10^ This finding emphasizes the necessity of avoiding or adopting extreme caution during sternal puncture in the lower part of the sternum.

### Clinical, radiological and forensic significance

SFs are usually asymptomatic and frequently detected incidentally on radiological images, surgical procedures, postmortem examinations and anatomy teaching specimens. ^1^^,^^2^^,^^4^^,^^5^^,^^10^ SF is a common variation that has been ironically referred to as “a finding of no clinical significance”. ^2^^,^^21^ Due to this, it is neglected in medical education, and only a few clinicians and acupuncturists are aware of SF. ^21^^,^^22^ The proximity of SF to vital adjacent thoracic structures constitutes a fundamental parameter with which clinicians and acupuncturists should be familiar. The iliac crest is the preferred site for bone marrow aspiration; however, extraction from the sternum becomes inevitable in certain circumstances, such as dry taps from the iliac crest or a history of pelvic radiotherapy. ^6^ On the other hand, the practice of acupuncture is considered inherently safe, but serious complications can sometimes occur. ^2^

In the scientific literature, 14 cases of cardiac and pericardial injuries have been reported during sternal puncture for bone marrow extraction or acupuncture in the lower sternal segment, of which 8 were fatal. A lack of awareness of the sternal foramen was the root cause of all fatal cases. ^2^ Therefore, during sternal interventions, avoidance of needle insertion in the lower thirds of the sternum and evaluation of previous radiographic scans or performing a preprocedural radiographic scan to rule out an SF are recommended.

Halvorsen et al. are to be commended for emphasizing the clinical significance of SF and for stressing the need for awaring medical students and clinicians during anatomy lessons, as well as on demonstrations of sternal puncture techniques. ^21^^,^^22^ In terms of acupuncture, the fourth and fifth CCJ levels correspond to commonly used acupuncture points and thus can pose great hazards when inserting acupuncture needles in persons with SF. The current study underpins the necessity for extra caution and avoidance of needle vertical insertion in the lower part of the sternum. ^2^^,^^6^ Therefore, an acupuncturist must use a superficial oblique insertion technique to prevent injury to thoracic organs in the study population.

Radiological evaluations can misdiagnose SF as lytic lesions or trauma. Therefore, it is vital for radiologists to be aware of this common variation and its radiographic characteristics to prevent misdiagnosis and subsequent mistreatment. When in doubt, radiologists should use computed tomography or magnetic resonance imaging, which best exposes the anatomical characteristics to enable discrimination, to differentiate SF from traumatic or osteolytic lesions of the sternum. ^2^^,^^3^^,^^6^

Furthermore, although SF is classically designated as a round or oval defect with a smooth margin, a study in Greece reported a single case of SF with an irregular margin and abnormal shape among five dried sternums with SF. ^3^ In our study, four (22.2%) SFs with irregular margins and abnormal shapes were observed among eighteen dried sternums with SF. Such features can considerably complicate the radiological differentiation of SF and osteolytic lesions due to their remarkable similarity. Therefore, radiologists should be aware of SF even with irregular margins and abnormal shapes. The presence of such lesions must be analyzed with caution, and SF must be considered in the differential diagnosis.

In this study, an extremely interesting case of a sternal foramen with a beveled margin was observed. To my knowledge, this is the first description of a sternal foramen with a beveled margin. However, in this case, the observed beveling is in complete contrast to the classical feature of a sternal foramen. This characteristic, if present, can considerably complicate forensic interpretation. In medicolegal postmortem examination of skeletonized human remains, SF can be misinterpreted as a projectile entry wound or other penetrating injury or even osteolytic lesion from cancer or infection. ^2^^,^^3^^,^^6^ If forensic experts and anthropologists are unaware of such anatomic variations, it may result in misclassification of the manner and cause of death. ^3^ SF is a midline defect, usually on the lower part of the sternum, with a round, oval or irregular shape, covered with cortical bone, and sometimes with a beveled margin.

Additionally, if such variations are routinely documented in the medical record whenever incidentally detected during the radiologic assessment, they can contribute to preprocedural evaluation before invasive sternal procedures. Furthermore, documentation of sternal bone variation is a simple and important potential source of data in the identification process of skeletonized human remains. However, there have been reports of perplexing similarities among members of the same family, highlighting the need for caution in interpretation. ^4^ Therefore, awareness of SF, together with routine documentation of such variations in antemortem medical and radiological records of individuals, can aid in accurate identification and investigation of the manner and cause of death.

### Xiphoid morphology

The term xiphoid process is derived from the Greek word “xiphos”, which means a straight sword that corresponds to the pointed end of the sternum. However, the xiphoid process can be absent, broad, bifid, trifurcated, duplicated or deflected. Variant xiphoid morphologies, such as bifid or trifurcated xiphoid processes, are caused by incomplete fusion of the ossification centers. ^2^^,^^5^ In the present study, bifid xiphoid processes occurred in 15.6% of cases, which is comparable to a study from Kenya. ^5^ In addition, trifurcated xiphoid processes were observed in a single (1.3%) specimen. Such variations could be misinterpreted as an epigastric mass, fracture, or traumatic fissure. ^1^^,^^5^ With increasing traffic-related injuries, clinicians should be aware of xiphoid variations to avoid misdiagnosis as a fracture or traumatic fissure. This necessitates special vigilance when assessing anterior chest wall injury, and it may therefore be considered a differential diagnosis. Furthermore, routine documentation of such variations, whenever detected incidentally, in antemortem medical records of individuals can aid in the identification of skeletonized human remains.

## Conclusions

The sternal foramen and variation in xiphoid morphology are common anatomical variations in Ethiopia. The findings of the current study highlight the necessity of strict precautionary measures during sternal procedures in this study population. Clinicians, radiologists, and anthropologists need to be aware of these variations, and their presence should be taken into consideration in everyday practice. Moreover, the presence of irregular and beveled sternal foramina considerably complicates radiological and anthropological differential diagnosis. Therefore, such incidental findings during radiologic and autopsy procedures should be properly evaluated to avoid misdiagnosis and misinterpretation of such findings as traumatic or pathological conditions.

## Data Availability

Figshare: Anatomical variations of the sternum: sternal foramen and variant xiphoid morphology in dried adult human sternum in Ethiopia, https://doi.org/10.6084/m9.figshare.23498958.v1. ^23^ This project contains the following underlying data:
- Anatomical variations of the sternum: sternal foramen and variant xiphoid morphology in dried adult human sternum in Ethiopia.xlsx Data are available under the terms of the Creative Commons Zero “No rights reserved” data waiver (CC0 1.0 Public domain dedication).
