# Supplementary material for: Anatomical variations of the sternum: sternal foramen and variant xiphoid morphology in dried adult human sternum in Ethiopia
Source: F1000Res. 2023 Aug 7;12:934. [Version 1] doi: 10.12688/f1000research.133151.1 (PMC11153988; doi:10.12688/f1000research.133151.1)
Supplement: Peer review history

# Reviewer report: Reviewer response for version 1

**DOI:** 10.5256/f1000research.146127.r276734

**Published:** 2024-06-05

**Authors:** Sven A Holcombe

**Affiliations:** 1 University of Michigan, Ann Arbor, Michigan, USA

The authors present a thorough investigation into the presence of sternal foramen (SF) in adults from a localized Ethiopian population. The sampling methodology is sound and statistical analysis appropriate. The manuscript is clear and well written. The authors find that their studied population has an SF incidence of ~19%, marginally higher than the previously described highest incidence (~18%). While this is remarked upon (as an outlier), as a reviewer I am not inclined to suspect a regional association with the presence of SF unless the data across multiple studies starts to show more concrete patterns and at higher population counts. (e.g., a chance change of a single observation from this study would put this study back within the range of previous studies).

 Note also that Table 1 lists the most recent paper's population (Bolatli 2020) as coming from an Australian source whereas it seems to instead be from Turkey. Please review this table for accuracy. This is another reason for my reluctance to see a regional difference in SF presence: data just from one country (Turkey) but different populations/methodologies ranges from 4.5-16.8% across multiple studies. I would suspect variation in selection criteria or examination technique before other biological factors.

 Other than that, just a few typos as suggested changes.

 Methods:

 Brocken -> Broken

 Results:

 The xiphoid process ended as a single in 83.1%

 In 15.6% (n=12/77) of the samples, the xiphoid process was bifurcated and trifurcated ...

 The xiphoid process was bifurcated in 15.6% (n=12/77) of the samples, and trifurcated ...

Is the work clearly and accurately presented and does it cite the current literature?

Yes

If applicable, is the statistical analysis and its interpretation appropriate?

Yes

Are all the source data underlying the results available to ensure full reproducibility?

Yes

Is the study design appropriate and is the work technically sound?

Yes

Are the conclusions drawn adequately supported by the results?

Yes

Are sufficient details of methods and analysis provided to allow replication by others?

Yes

Reviewer Expertise:

Computational anatomy and biomechanics

I confirm that I have read this submission and believe that I have an appropriate level of expertise to confirm that it is of an acceptable scientific standard.

# Reviewer report: Reviewer response for version 1

**DOI:** 10.5256/f1000research.146127.r230958

**Published:** 2024-05-25

**Authors:** Holger Wittig

**Affiliations:** 1 Institute of Forensic Medicine, University Basel, Basel, Switzerland

Scientific papers on morphological variations of the sternum are rare. Therefore, it is welcoming that this paper reports variations of the sternum in an Ethiopian population.

 The introduction mentions the high variability and, in the forensic context, its application for identification. However, corresponding references regarding identification are missing in the literature, such as:

 (Weiss G et al,2018) [Ref 1] or

 (Verna, E. et al, 2013) [Ref 2] or

 (Leite, V. M., et al,2020) [Ref 3]

 The statistical analysis of the data is perplexing. Presenting an average age with four decimal places (38.383 ± 11.3840 years) implies a level of precision that may be misleading and is highly uncommon. Age should not be specified beyond one decimal place, and a better choice would be to provide the median as a measure of central tendency. Foramina in the sternum result from incomplete fusion of sternal ossification centers during embryogenesis. Therefore, foramina in the sternum are congenital. Reporting significant age differences in the frequency of foramina can only be a random pseudo-result due to sampling errors.

 The quality of the photos is poor. The photos are unevenly illuminated (figures 2 and 3), the background is unclean (figures 1, 3, 5), and figure 4 appears blurry. Post-processing of the photos (black and white, background cleaning) or the use of scientific drawings is strongly recommended. Conventional X-ray images would also be an option.

 The statement asserting that variations in the sternum could contribute to determining the manner and cause of death lacks explanation. One can imagine that this might be possible in gunshot or stab injuries. However, anatomical variations seldom explain the cause of death.

 Especially in the Discussion section, there are many redundant, partially unsupported statements. The section should be significantly shortened.

 The epidemiological data on morphological variations is welcomed. However, in its current form, a substantial revision of the paper is needed.

Is the work clearly and accurately presented and does it cite the current literature?

Partly

If applicable, is the statistical analysis and its interpretation appropriate?

No

Are all the source data underlying the results available to ensure full reproducibility?

Yes

Is the study design appropriate and is the work technically sound?

Partly

Are the conclusions drawn adequately supported by the results?

Partly

Are sufficient details of methods and analysis provided to allow replication by others?

Yes

Reviewer Expertise:

general forensic pathology, forensic anthropology, forensic radiology

I confirm that I have read this submission and believe that I have an appropriate level of expertise to confirm that it is of an acceptable scientific standard, however I have significant reservations, as outlined above.

## References

[1] : Identification of deceased based on sternal bone computed tomography features. *Forensic Sci Int* .2018;286: 10.1016/j.forsciint.2018.03.018 233-238 10.1016/j.forsciint.2018.03.018 29602150

[2] : Discrete traits of the sternum and ribs: a useful contribution to identification in forensic anthropology and medicine. *J Forensic Sci* .2013;58(3) : 10.1111/1556-4029.12111 571-7 10.1111/1556-4029.12111 23550726

[3] : Sternal Variation: Anatomical-Forensic Analysis. *International Archives of Medicine* .2020;13: 10.3823/2626 10.3823/2626

# Reviewer report: Reviewer response for version 1

**DOI:** 10.5256/f1000research.146127.r260376

**Published:** 2024-04-03

**Authors:** Andrea Sonaglioni

**Affiliations:** 1 IRCCS MultiMedica, San Giuseppe Hospital, Milan, Italy

The Authors provided baseline epidemiological information about the sternal foramen (SF) and variant xiphoid morphology in Ethiopia, comparing results with other populations and discussing the clinical and forensic significance. 

 The Authors exhaustively described all the anatomical variants and clinical characteristics.of SF.

 The Authors reported an incidence of SF in the Ethiopian population of 19.1%; sternal foramina were more common in men than in women.

 Given that SF is a common variation that has been ironically referred to as “a finding of no clinical significance", it is usually neglected in medical education, and only a few clinicians and acupuncturists are aware of SF. However, it is important to differentiate SF from traumatic or osteolytic lesions of the sternum. Moreover, from a clinical point of view, when inserting acupuncture needles, given that the sternal foramen is more commonly localized in the lower part of the sternum, it is important to avoid a needle vertical insertion in the lower part of the sternum. An oblique insertion technique may prevent injury to thoracic organs". 

 I have only a suggestion for the Authors.

 In the Discussion section, after the sentence: "An oblique insertion technique may prevent injury to thoracic organs", the Authors could also add the potential usefulness of an echocardiographic monitoring during the needles' insertion procedure. In fact, the transthoracic parasternal long-axis view might be particularly useful for assessing the antero-posterior thoracic diameter, measured from the true apex of the sector (the point of entry of ultrasound) and the spine, the latter visualized behind the posterior wall of the descending aorta. The cardiac chambers are all visualized within this simple echocardiographic section and their integrity may be verified during the needles' insertion procedure.

 The Authors could cite following reference: Sonaglioni A, et. al., 2018 (Ref 1)

Is the work clearly and accurately presented and does it cite the current literature?

Yes

If applicable, is the statistical analysis and its interpretation appropriate?

Yes

Are all the source data underlying the results available to ensure full reproducibility?

Yes

Is the study design appropriate and is the work technically sound?

Yes

Are the conclusions drawn adequately supported by the results?

Yes

Are sufficient details of methods and analysis provided to allow replication by others?

Yes

Reviewer Expertise:

Echocardiography

I confirm that I have read this submission and believe that I have an appropriate level of expertise to confirm that it is of an acceptable scientific standard, however I have significant reservations, as outlined above.

## References

[1] : A New modified anthropometric haller index obtained without radiological exposure. *Int J Cardiovasc Imaging* .2018;34(10) : 10.1007/s10554-018-1366-5 1505-1509 10.1007/s10554-018-1366-5 29766334
